# Construction and Enhancement of a Rural Road Instance Segmentation Dataset Based on an Improved StyleGAN2-ADA

**DOI:** 10.3390/s25082477

**Published:** 2025-04-15

**Authors:** Zhixin Yao, Renna Xi, Taihong Zhang, Yunjie Zhao, Yongqiang Tian, Wenjing Hou

**Affiliations:** 1 College of Computer and Information Engineering, Xinjiang Agricultural University, Urumqi 830052, China; 320192868@xjau.edu.cn (Z.Y.); xren@xjau.edu.cn (R.X.); 320200031@xjau.edu.cn (Y.Z.); hwj@xjau.edu.cn (W.H.); 2 Research Center for Intelligent Agriculture, Ministry of Education Engineering, Urumqi 830052, China; 3 Xinjiang Agricultural Informatization Engineering, Technology Research Center, Urumqi 830052, China

**Keywords:** rural road, instance segmentation, StyleGAN, data augmentation, image generation

## Abstract

With the advancement of agricultural automation, the demand for road recognition and understanding in agricultural machinery autonomous driving systems has significantly increased. To address the scarcity of instance segmentation data for rural roads and rural unstructured scenes, particularly the lack of support for high-resolution and fine-grained classification, a 20-class instance segmentation dataset was constructed, comprising 10,062 independently annotated instances. An improved StyleGAN2-ADA data augmentation method was proposed to generate higher-quality image data. This method incorporates a decoupled mapping network (DMN) to reduce the coupling degree of latent codes in W-space and integrates the advantages of convolutional networks and transformers by designing a convolutional coupling transfer block (CCTB). The core cross-shaped window self-attention mechanism in the CCTB enhances the network’s ability to capture complex contextual information and spatial layouts. Ablation experiments comparing the improved and original StyleGAN2-ADA networks demonstrate significant improvements, with the inception score (IS) increasing from 42.38 to 77.31 and the Fréchet inception distance (FID) decreasing from 25.09 to 12.42, indicating a notable enhancement in data generation quality and authenticity. In order to verify the effect of data enhancement on the model performance, the algorithms Mask R-CNN, SOLOv2, YOLOv8n, and OneFormer were tested to compare the performance difference between the original dataset and the enhanced dataset, which further confirms the effectiveness of the improved module.

## 1. Introduction

With the rapid advancement of autonomous driving technology, the application of agricultural machinery autonomous driving systems in rural road environments has become increasingly important. Machine vision and autonomous navigation [1,2] technologies have emerged as focal points of research. Deep learning [3], owing to its high precision, strong robustness, and relatively low cost, has made significant strides in the field of autonomous driving. However, the effective training and superior performance of instance segmentation models largely depend on datasets that are abundant in samples and evenly distributed. Currently, most existing road instance segmentation datasets, such as Cityscapes, BDD100k, and Mapillary Vistas, primarily focus on urban road scenarios [4,5], with relatively less coverage of rural roads, unstructured road sections, and special environments like farmlands and woodlands. These existing datasets fall short of meeting the needs of rural road autonomous driving systems for training instance segmentation models. Therefore, constructing a high-quality dataset that comprehensively covers the characteristics of rural roads is not only crucial for enabling intelligent agricultural machinery to achieve autonomous navigation, precise obstacle avoidance, and safe operation, but also provides a solid data foundation for advancing the intelligent construction of rural areas.

To enhance the diversity of data samples and the generalization capability of models, data augmentation has become an effective strategy. Data augmentation not only increases the quantity of samples but also improves the robustness and generalization of models by introducing environmental variations such as different weather conditions, lighting, and roadside vegetation. Additionally, data augmentation can effectively address potential sample imbalance issues in rural road datasets, thereby enhancing the model’s ability to recognize minority classes. Image-based data augmentation methods [6] can be categorized into traditional image processing techniques and machine learning-based [7] approaches. Traditional data augmentation methods include geometric transformations, random adjustments of brightness and contrast, and the addition of various types of noise to the original images. Kumar et al. [8] evaluated the impact of traditional and deep learning techniques on common computer vision tasks (image classification, object detection, and semantic segmentation) through a comprehensive study of image data enhancement techniques, which can be more effective in improving the accuracy of the classification algorithms as compared to the traditional data enhancement methods based on deep learning. The use of generative adversarial networks (GANs) [9] for data augmentation has become a focal point of current research. These methods generate new, realistic data samples by learning the underlying distribution of the data, thereby increasing the diversity and size of the dataset. For example, Yang et al. [10,11,12] proposed a CycleGAN network incorporating structural constraint loss, self-attention mechanisms, and pre-registration strategies. This method achieved high-fidelity anatomical geometry in unsupervised MR-to-CT synthesis, producing CT images with high structural consistency that can be directly used for dose calculation, reducing the model’s reliance on real CT images. This not only provides a reliable synthetic imaging tool for precision radiotherapy but also offers a new optimization framework for cross-modal medical image generation tasks. Wu et al. [13,14] addressed the issue of tomato leaf disease recognition by proposing a deep convolutional generative adversarial network (DCGAN). By optimizing the GAN architecture, tuning hyperparameters, and improving convolutional neural network design, they validated the realism and diversity of the generated images using t-distributed stochastic neighbor embedding (t-SNE) for dimensionality reduction visualization and visual Turing tests, effectively tackling data scarcity in small-sample scenarios. Karras et al. [15] introduced StyleGAN, building upon GANs with a unique architectural design capable of generating high-quality, high-resolution images and offering unprecedented flexibility in controlling specific styles and content within the images.

CycleGAN and DCGAN have demonstrated significant advantages in unsupervised cross-modal image generation and small-sample scenarios, while StyleGAN, with its unique architectural design, has achieved the generation of high-quality, high-resolution images and offers flexible control over image style and content. However, for image generation and data augmentation tasks in rural road environments, two key challenges remain:

(1) Coupling Effect in W-Space Latent Codes: The single mapping network employed in the existing StyleGAN2-ADA model often leads to strong coupling effects among the dimensions of the latent codes in the W-space, thereby limiting the fine-grained control over visual styles of different categories.

(2) Limitations of Traditional Convolutional Modules: Traditional convolutional modules are insufficient in capturing global contextual information and long-range dependencies, making it difficult to meet the demands of high-resolution complex scenes.

To address these challenges, this paper proposes an improved StyleGAN2-ADA-based image data augmentation method, aiming to generate more diverse and richer rural road image data and enhance the recognition accuracy of instance segmentation models. The specific improvements include the following three aspects:

(1) Decoupled Mapping Network (DMN): The mapping network in the original model is improved, and the original eight-layer fully connected mapping network is divided into four groups of sub-networks; each group contains two fully connected layers. Each group of sub-networks focuses on different features. Through DMN ablation experiments, it is verified that the design can effectively reduce the coupling between each dimension of w-space implicit coding, realize the fine regulation of various visual styles, and generate images with more diverse styles and richer details.

(2) Convolutional Coupling Transfer Block (CCTB) [16]: To address the limitation of convolutional modules in extracting only local features, the CCTB module is designed by combining the strengths of convolutional neural networks (CNNs) and transformers. By introducing the cross-shaped window self-attention mechanism (CSWin-Attention), the module efficiently captures long-range dependencies in images while maintaining the convolutional network’s ability to extract local details. This enhances the perception of complex contextual information and spatial layouts.

(3) Ablation Study Validation: In this paper, two sets of ablation experiments are designed to verify the decoupling ability of DMN, and the use of DMN and CCTB modules can improve the quality and diversity of generated images. The combined StyleGAN-ALL model achieves significant advantages in generation quality, edge clarity, and performance on downstream instance segmentation tasks. On four different network models of Mask R-CNN [17], SOLOv2 [18], YOLOv8n [19], and OneFormer [20], the original dataset and the fusion dataset enhanced by the improved data are used for training, and the effectiveness of each improved module is verified through qualitative and quantitative evaluation. The experimental results fully prove the innovative contribution of the improved method in dealing with rural road image generation.

## 2. Materials and Methods

### 2.1. Data Acquisition

#### 2.1.1. Data Categories

This study aims to establish a data foundation for smart agricultural machinery technology in agricultural production bases. The data collection area is set in typical scenarios such as rural areas, fields, and urban–rural roads. The data collection work systematically covers various typical road types, including asphalt roads, cement roads, gravel roads, and dirt roads, based on the temporal and spatial characteristics of agricultural machinery operations. Special emphasis is placed on two representative time periods for image collection: daytime (with sufficient lighting) and dusk (with changing lighting conditions). The object categories for image recognition mainly include dynamic objects (such as agricultural machinery vehicles, cars, trucks, tricycles, bicycles, pedestrians, and livestock) and static objects (such as trees, traffic signs, streetlights, fences, walls, and other infrastructure).

#### 2.1.2. Data Collection Equipment

**Figure 1:**
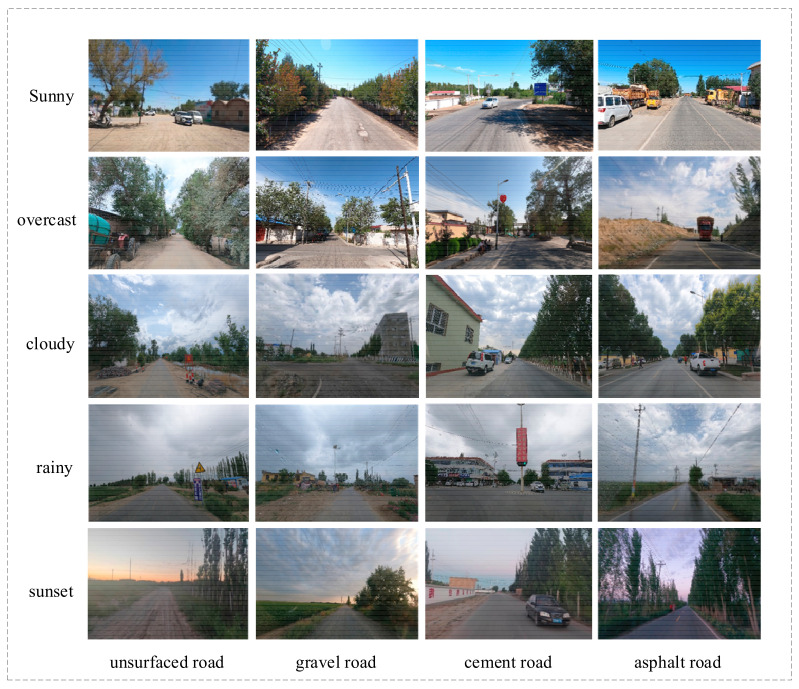
Image collection scenario.

In this study, a GoPro HERO9 monocular sports video camera (Gropro Inc., Etna, CA, USA) was used as the primary data collection device. This device features a resolution of 3840 × 2160 pixels, a frame rate of 30 frames per second, and supports 5K video recording and a 20-megapixel photo capture. Equipped with HyperSmooth 3.0 video stabilization and built-in horizon leveling, it ensures the stability and continuity of image acquisition. Additionally, a smartphone with 4K/30 fps high-definition video recording capabilities was employed as a supplementary collection device. During the data collection process, the imaging devices were mounted on the car’s rearview mirror, and data was collected at a constant speed of 30 km/h, accumulating 6 h of video data. The video data was processed using frame extraction techniques, followed by rigorous image screening and preprocessing, resulting in a final dataset of 1285 valid image samples. The specific collection scenario is illustrated in Figure 1.

#### 2.1.3. Data Annotation

In this study, the Computer Vision Annotation Tool (Intel Corporation, Santa Clara, CA, USA) was employed to set up a multi-user online image annotation platform on a local server. The polygon annotation tool was utilized to perform pixel-level fine-grained annotation on all images. The dataset constructed in this study contains 39 categories, 20 of which have instance segmentation annotations. The categories of instances can be grouped into six broad categories, which are as follows: **animal** (animal), **block** (barrier and banner), **person** (person), **objects** (CCTV camera, street light, trash can, tower, and pole), **traffic signaling device** (traffic frame, traffic light, and traffic sign), and **vehicle** (car, bus, motorcycle, truck, rickshaw, agricultural implements, agricultural machinery, and tricycle). The other 19 classes (such as vegetation, sky, building, etc.) are semantic segmentation categories, and related research will be carried out in another paper. The 20 categories listed in this paper only cover the instance segmentation part, which is mainly used to evaluate the compatibility of the segmentation performance of synthetic images at the object level with real images to support the modeling requirements of subsequent obstacle avoidance tasks.

**Figure 2:**
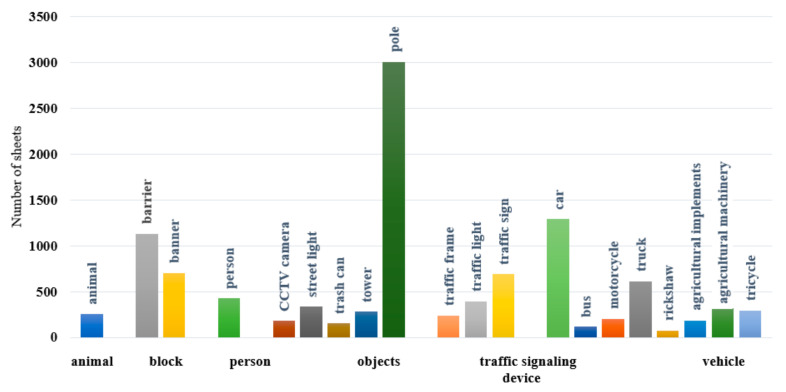
Statistical chart of instance category quantities.

During the annotation process, all instances were annotated with non-overlapping boundaries to ensure the accuracy and completeness of the annotations. A total of 10,062 instances were annotated. Among these, the **objects** category had the highest number of instances, totaling 3973, with an average of approximately 3 instances per image. This was followed by the **vehicle** category, which included 3126 instances, averaging about 2 instances per image. The **animal** category had the fewest instances, with only 256 annotated. The distribution of instance counts across categories is illustrated in Figure 2.

**Figure 3:**
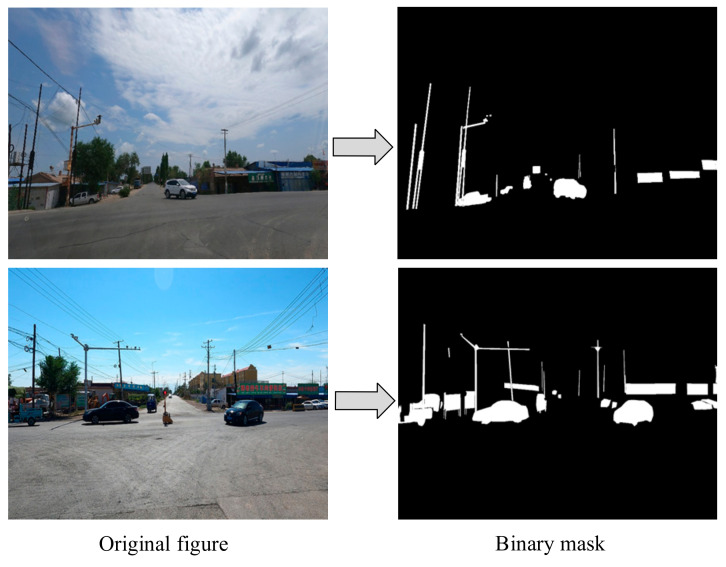
Example comparison of original images and mask Images.

Instance segmentation is a pixel-level image segmentation task with the core objective of distinguishing each individual object instance in an image beyond semantic segmentation and generating a unique mask for each instance. To achieve this goal, this study utilized the Python 3.7 programming language to parse the instance annotation information stored in JSON files, generating mask images corresponding to each original image. Specifically, based on the polygon vertex coordinates provided in the annotations, the pixel regions of each instance were precisely mapped to the mask images, with unique identifier values assigned to different instances. An example of the generated mask images is shown in Figure 3, which clearly demonstrates the independent segmentation results for each instance.

### 2.2. Data Augmentation Methods

**Figure 4:**
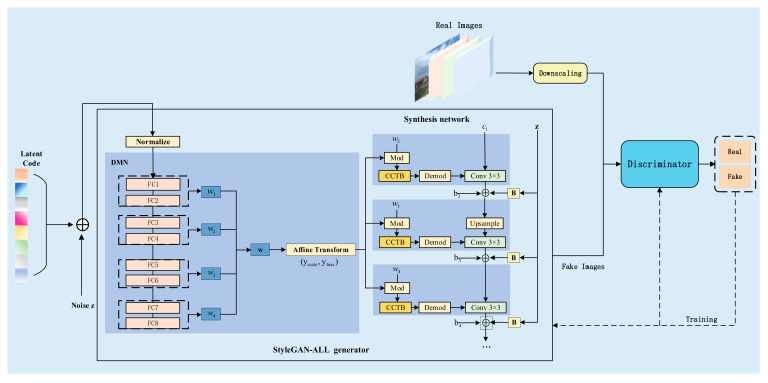
Structure diagram of the improved StyleGAN-ALL network.

The exceptional performance of generative adversarial networks (GANs) in image generation tasks primarily relies on the powerful nonlinear fitting capabilities of neural networks. Consequently, the quality and diversity of generated images are directly influenced by the design of the network architecture [21]. To solve the problem of limited dataset scale in this study and provide richer data samples for the training of subsequent instance segmentation models, an improved model, StyleGAN-ALL, based on StyleGAN2-ADA, is proposed in this study, and the basic structure of the network is shown in Figure 4. This model further optimizes the quality and diversity of generated images by introducing the decoupled mapping network (DMN) and the convolutional coupling transfer block (CCTB). Specifically, the decoupled mapping network reduces the coupling degree of latent space encoding, and the CCTB module enhances the model’s ability to capture complex context information by combining the advantages of convolutional neural networks (CNNs) and transformers.

#### 2.2.1. StyleGAN2-ADA Network Model Architecture

The core idea of the traditional GAN network is to make the generator (G) and the discriminator (D) compete with each other through adversarial training so that the generator generates realistic data and fools the discriminator. The generator outputs data, such as images and texts, by inputting a random noise vector z. The discriminator inputs real data x or generated data G(z), and outputs a probability value (0~1) to indicate the likelihood that the data are real data. The training process is a Minimax Game process, and the optimization objective function is as follows.

(1) minGmaxDVD,G=Ex~pdatalogD(x)+Ez~pzlog⁡(1−DGz)

StyleGAN is NVIDIA’s (NVIDIA Corporation, Santa Clara, California) improved GAN model for generating high-resolution and high-quality images. His innovations include Mapping Network and Adaptive Instance Normalization (AdaIN). The main process is to map the random noise vector z to the intermediate latent space w, and generate the latent code with more semantic and structural information through the mapping network f (composed of 8 fully connected layers) and then generate the style parameters $y_{scale}$ and $y_{bias}$ through affine transformation. It is used to perform Adaptive Instance Normalization on the features of each layer of the generator. The mathematical formula for AdaIN is as follows.

(2) $$AdaIN\left(x,y\right)=y_{scale}\cdot \frac{x-\mu \left(x\right)}{\sigma \left(x\right)}+y_{bias}$$

Here, x is the activation feature of the current layer, and $\mu \left(x\right)$ and $\sigma \left(x\right)$ denote the mean and standard deviation of x, respectively. Through the design of this module, the model can fine-tune the image at different scales, but the shortcomings are that premature statistical normalization will lose local details and produce artifacts, and the source of each dimension’s hidden coding output of the mapping network is too singular, so there is a strong coupling effect. In view of the above problems, it is found that AdaIN and progressive training are the main reasons for the abnormalities of the generated images. Therefore, StyleGAN2 proposes a novel modulated convolution mechanism to solve the Droken artifacts problem by replacing the AdaIN module with modulation and demodulation. It is calculated as follows:

(3) $$\left\{\begin{array}{c} W_{ijk}^{\prime }=s_{i}\cdot W_{ijk}\\ W_{ijk}^{\prime \prime }=\frac{W_{ijk}^{\prime }}{\sqrt{\sum_{i,k}{\left(W_{ijk}^{\prime }\right)}^{2}+\epsilon }} \end{array}\right.$$

Here, $s_{i}$ is the modulation factor for input channel I, which can scale the input channel. $W_{ijk}^{\prime }$ is the modulated weight. $W_{ijk}^{\prime \prime }$ is the normalized demodulated weight of $W_{ijk}^{\prime }$ grouped by output channel j. Its role is to constrain the variance of the output feature map to be 1, so as to avoid the instability of the feature amplitude caused by modulation. The ϵ is a minimal constant, preventing the denominator from being zero. At the same time, we introduce path length regularization (PLR) to constrain the change of the latent space w to be linearly related to the change of the generated image so that the gradient length of the generator is relatively stable in all directions, which is calculated as follows:

(4) $$E_{w\sim f\left(z\right),y\sim N(0,Ι)}\left[{\left({\left\|\nabla_{w}({G\left(w\right)}^{T}y)\right\|}_{2}-a\right)}^{2}\right]$$

Here, $w\sim f\left(z\right)$ means that the latent code w is generated by the mapping network f from the noise z (that is $w=f\left(z\right)$). y~N(0,Ι) means that the random vector y is sampled following a standard Gaussian distribution and is used to project the generated image $G\left(w\right)$. $\nabla_{w}({G\left(w\right)}^{T}y)$ is the gradient in a random direction y of the generated image $G\left(w\right)$ after derivative with respect to w. a is a hyperparameter and represents the object path length.

StylegGAN2-ADA [22,23] is a further improvement on StyleGAN2, which mainly focuses on the problem that the discriminator is easy to overfit in the training process of small datasets. The core innovations are the adaptive data augmentation (ADA) strategy and the improved discriminator training mechanism. In the training process, data augmentation operations such as flipping, rotation, and color jitter are performed on the input images, and the enhancement probability p is dynamically adjusted according to the overfitting of the discriminator. The adaptive enhancement probability formula is as follows:

(5) $$\left\{\begin{array}{c} r_{t}=E\left[sign(D_{train})\right]-E\left[sign(D_{validation})\right]\\ p_{t+1}=p_{t}+\eta \cdot clip(r_{t}-r_{target},-c,c) \end{array}\right.$$

where $r_{t}$ is the overfitting index, and the larger the value, the more serious the overfitting is. $D_{train}$ is the discriminator’s prediction on the training set (augmented), and $D_{validation}$ is the discriminator’s prediction on the retained true images (unaugmented). $p_{t+1}$ is the augmented probability after updating, η is the learning rate, $r_{target}$ is the object overfitting level, and clip(⋅) is the limit change to avoid sharp p fluctuations.

However, this model still has some shortcomings. Although ADA alleviates the small dataset problem to some extent, generative models may still struggle to capture rich style variation in very scarce data scenarios. Therefore, the quality and diversity of the generated images are optimized for further optimization. We introduce the convolutional coupling transfer block (CCTB) in our improved model StyleGAN-ALL proposed in this study. The CCTB module is inserted in the middle of modulation and demodulation, as shown in the orange module in Figure 4. This module combines the advantages of convolutional neural networks (CNNs) and transformers, which can effectively enhance the model’s ability to capture complex context information. In addition, the coupling effect of w-space implicit coding in the original design may limit the independent control of diverse styles, which is why we tried to improve by decoupling the mapping network in the follow-up study. To solve this problem, a decoupled mapping network (DMN) is proposed in Section 2.2.2 in this study to reduce feature coupling by grouping design.

#### 2.2.2. Decoupled Mapping Network (DMN)

Since the features of each dimension of w-space in the original design are generated by the same mapping network, there may be coupling effects between different style features, which may affect the fine control of diversified image generation. In view of the diversity of category styles contained in the rural road dataset, it is necessary to reduce this coupling in order to better realize the fine generation of independent styles of each category. In this study, we improved the mapping network based on this model by dividing the original 8-layer fully connected (FC) mapping network into four groups of independent subnetworks $\left\{g_{1},g_{2},g_{3},g_{4}\right\}$, which are calculated as shown in Formula (6). Each group of subnetworks contains two layers of FC, and each group outputs an intermediate latent code $w_{i}\in R^{m/4}$. Finally, the outputs of the four layers are concatenated into a complete w. Each group of sub-networks focuses on different features, $w_{1}$ corresponds to road structure and geometric layout, such as straight, curved, fork roads, and terrain relief. $w_{2}$ corresponds to the natural environment and vegetation, such as vegetation types on both sides of the road, vegetation density, and vegetation wilting due to seasonal changes. $w_{3}$ corresponds to weather and lighting conditions, such as sunny days, cloudy days, rainy days, early morning, evening, etc., and the lighting direction and shadow position can be changed by adjusting $w_{3}$. $w_{4}$ corresponds to artificial objects, such as road signs, surrounding buildings, utility poles, fences, etc. These latent encodings are then used to modulate the convolution kernels of each convolutional layer in the generator after their respective affine transformations (i.e., the modulation convolution and demodulation mechanism are employed). To verify the effectiveness of the proposed method, we added a set of DMN ablation experiments to the experimental section. It is found that the DMN effectively reduces the strong coupling relationship between w-space implicit coding, so as to promote the generation network to generate images with more diversity and higher style discrimination.

(6) $$g_{i}\left(z\right)=W_{i}^{\left(2\right)}\sigma \left(W_{i}^{\left(1\right)}z+b_{i}^{\left(1\right)}\right)+b_{i}^{\left(2\right)}$$

The $W_{i}^{\left(l\right)}$ and $b_{i}^{\left(l\right)}$ denote the group I first l layer weights and bias, σ denotes the activation function, and the final latent code w represents each output, namely $w=\left[w_{1};w_{2};w_{3};w_{4}\right]=\left[g_{1}\left(z\right);g_{2}\left(z\right);g_{3}\left(z\right);g_{4}(z)\right]$.

#### 2.2.3. Convolutional Coupling Transfer Block (CCTB)

In StyleGAN2-ADA, the feature learning modules at each layer still rely on basic convolutional modules. However, convolutional modules have limited receptive fields and can only extract local information from the multi-scale features progressively generated by the generator. This results in suboptimal performance in terms of continuity and generation quality when producing images with strong semantic information. Particularly in rural road image datasets, the generation of objects such as roads, trees, and skies depends on the perception of long-range dependencies. Compared to convolutional modules, transformers [24,25] exhibit stronger capabilities in modeling long-range dependencies, making them more suitable for generating such objects. Nevertheless, transformers also have certain limitations, such as slower convergence rates and the need for large amounts of training data. Therefore, to combine the strengths of both CNNs and transformers, this study designs a convolutional coupling transfer block (CCTB).

**Figure 5:**
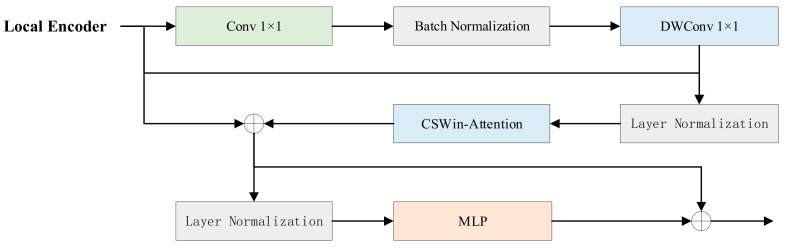
CCTB module structure diagram.

The module is primarily divided into five components: a local encoding module (LEM), a cross-shaped window self-attention (CSWin-Attention), a batch normalization (BN), a layer normalization (LN), and a multilayer perceptron (MLP). Its structure is illustrated in Figure 5. The computational process of the CCTB module is as follows:

(7) $${Ζ}_{l}^{\prime }=CSWin-Attention\left(LN\left(LEM\left(Z_{l-1}\right)+Z_{l-1}\right)+Z_{l-1}\right)$$

(8) $$Z_{l}=MLP\left(LN\left(Z_{l}^{\prime }\right)\right)+Z_{l}^{\prime }$$

where ${Ζ}_{l}^{\prime }$ represents the intermediate output after applying the CSWin-Attention mechanism to the normalized features from the LEM. It captures enhanced contextual information by combining local features and long-range dependencies. $Z_{l}$ is the final output of the CCTB module at layer l, representing a comprehensive feature map that combines local and global information.

1. Local Encoding Module (LEM)

In image processing tasks of rural road scenes, the variation of object appearance and location often poses a significant challenge for model recognition. Especially, the boundaries between objects in such unstructured scenes are usually not obvious, presenting fuzzy characteristics. In order to solve this problem, combining the dual advantages of CNNs and transformers is an important move. Firstly, CNNs can have a robust local feature extraction ability through a local receptive field and weight-sharing mechanism so as to effectively identify the edge information of these objects. Second, transformers incorporates position-specific encodings for each object to represent their relative position, which enhances the model’s robustness to translation, scaling, and distortion.

To achieve effective fusion of local details and global context, LEM is used to extract fine-grained local features. Then, these local features are fused with the global feature map generated by transformers through residual connection for multi-scale fusion. The fusion process is shown in Formula (9). It not only retains the sensitivity of CNN to local structural information but also integrates the long-distance dependence modeling ability of transformers and, finally, forms a comprehensive feature representation containing local details and global context. This composite feature representation mechanism improves the robustness of the model to appearance changes and location shifts of objects in complex rural road scenes.

(9) $$LEM\left(Z_{l}\right)=DWConv\left(BN\left(Conv\left(Z_{l}\right)\right)\right)+Z_{l}$$

where DWConv represents the depthwise separable convolution.

2. Cross-Shaped Window Self-Attention (CSWin-Attention)

**Figure 6:**
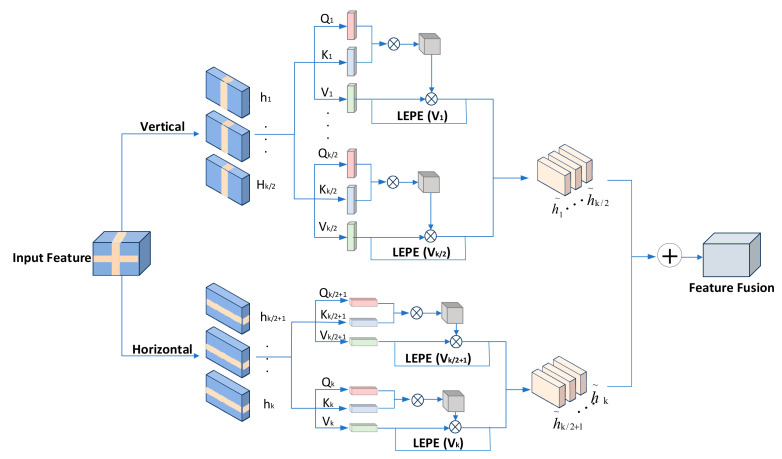
Cross-shaped window self-attention structure diagram.

Rural road scenes usually contain diverse objects, such as trees, traffic signs, and vehicles. The spatial relationships between these objects are complex, and there may be partial occlusions or uneven distributions. Traditional convolutional operations have limited receptive fields, making it difficult to capture global contextual information. The CSWin-Attention module divides the feature map into non-overlapping vertical and horizontal windows through dynamic window offset at different levels and randomly offsets $△\in \left[0,sw/2\right]$ pixels (sw is the window size). Through multi-level CCTB stacking, the dependence of the cross region can be progressively modeled. Thus, the problem of artifacts in vegetation interleaved areas can be alleviated. The shallow network with a dynamic window offset compensation mechanism has a small Δ value, which preserves local details (such as vegetation leaf texture), and the deep network has an increased Δ value, which can model the global semantic association of intersection regions (such as the topological relationship between vegetation and roads). Through multi-level concatenation stacking, the context information of the cross region is gradually fused to avoid feature breakage caused by fixed window division. In addition, CSWin-Attention uses multi-head attention grouping, which assigns part of the attention heads to the vertical window and the other part to the horizontal window so that the model models the context of different directions at the same time. This combination covers the intersection area at the junction of the two windows, reducing the artifacts caused by single-direction attention misses. Moreover, because the long-distance dependence modeling ability of a transformer is integrated, the features of the intersection area can be more reasonably modeled at different scales so as to better understand the spatial layout and semantic association between objects. It improves the overall coherence of the generated image. Additionally, rural road images often have high resolution, and traditional global self-attention mechanisms [26] have high computational complexity, making them difficult to apply directly to such scenarios. CSWin-Attention reduces computational complexity significantly by dividing the feature map into windows and computing self-attention within these windows. At the same time, the cross-shaped window design, which combines horizontal and vertical computations, ensures the capture of global information, achieving a balance between efficiency and performance in high-resolution image processing. This mechanism splits the input feature map $Ζ\in R^{C\times H\times W}$ along the channel dimension into horizontal and vertical attention branches, allowing independent computation of attention weights in both directions. The main structure of this mechanism is shown in Figure 6.

Vertical attention is able to capture long-distance dependence in vertical directions, helping the model understand the up-down relationship between sky and ground, trees and roads. It is calculated by dividing the input feature map $Ζ\in R^{C\times H\times W}$ into multiple non-overlapping vertical bands $\left\{{Ζ}^{1},{Ζ}^{2},\cdots ,{Ζ}^{Μ}\right\}$ along the vertical direction, where the width of each vertical band is sw and M=H/sw. The index range for each vertical band is defined as follows:

(10) $${Vertical\;Band}_{j}=Z\left[:,\;:,\left(j-1\right)\cdot sw+△:j\cdot sw+△\right]$$

Then we apply a linear projection to each vertical band ${Ζ}^{j}$ to obtain the input matrix for self-attention, as shown in the following formula:

(11) $$\left\{\begin{array}{c} Q_{j}={Ζ}^{j}W_{Q}\\ K_{j}={Ζ}^{j}W_{K}\\ V_{j}={Ζ}^{j}W_{V} \end{array}\right.$$

where $Q_{j}$, $K_{j}$, and $V_{j}$ correspond to queries, keys and values, respectively, and $W_{Q}$, $W_{K}$, and $W_{V}$ are learnable weight matrices. Through Formula (11), the following self-attention weight calculation formula in the vertical direction can be obtained:

(12) $$A\left(Q_{j},K_{j},V_{j}\right)=Softmax\left(\frac{Q_{j}K_{j}^{T}}{\sqrt{d_{k}}}\right)V_{j}+LEPE(V_{j})$$

where $d_{k}$ is the dimension of the key vector and $LEPE(V_{j})$ is the local position encoding implemented by depthwise separable convolution.

Horizontal attention primarily focuses on the semantic associations of images in the horizontal direction. It not only captures dependencies between left and right adjacent regions, helping the model understand the extension direction of roads and the arrangement order of vehicles in rural image datasets but also enhances global context information. By computing horizontal attention, the model can better understand the distribution and interactions of objects in the horizontal direction, such as the relative positions between vehicles and pedestrians. Its calculation method is the same as that of vertical attention, and the final output feature map $Z_{out}$ is obtained through the weighted fusion of vertical and horizontal attention.

(13) $$Z_{out}=\alpha \cdot Z_{ver}+(1-\alpha )\cdot Z_{hor}$$

where $Z_{ver}$ and $Z_{hor}$ are the outputs of vertical attention and horizontal attention, respectively, which are obtained by globally aggregating the local self-attention results $A\left(Q_{j},K_{j},V_{j}\right)$. ∝∈[0,1] is the learnable fusion weight, initialized with a value of 0.5 and automatically optimized by backpropagation, which is used to balance the contributions of vertical and horizontal attention in the final output. In addition, the fusion method of Formula (13) can have an indirect effect on the diagonal direction. When the weights ∝ and 1−∝ take different proportions, they form feature combinations of different directions in the feature space. For example, when ∝ is 0.6 and 1−∝ is 0.4, then the synthesis direction and diagonal direction $\theta =arctan\frac{0.6}{0.4}\approx 56.3^{\circ }$, so it has a certain diagonal information capture ability.

The fusion of vertical and horizontal attention enables comprehensive modeling of complex scenes, effectively addressing issues such as uneven object distribution, occlusion, and multi-scale feature fusion in rural road image datasets. This provides robust support for image generation and instance segmentation tasks, particularly in high-resolution image processing scenarios.

## 3. Experiments

### 3.1. Experimental Environment and Parameter Settings

In order to improve the generalization ability of the model under the condition of a small sample, this study adopts the transfer learning strategy. Firstly, the network model is pre-trained for 100 epochs on the public datasets Mapillary Vistas and BDD100K so that the model can fully learn the diverse semantic features in the vehicle driving scene. Among them, Mapillary Vistas has high-quality panoramic annotation covering multiple road environments. BDD100K provides a rich driving visual scene and enhances the model’s ability to understand traffic elements. After the pre-training is completed, the model is transferred to our self-built rural road panoramic segmentation dataset for fine-tuning so as to achieve effective adaptation and optimization for specific scenes. Our dataset focuses on rural road scenes, which are generally missing in existing mainstream datasets. The data covers a variety of typical rural characteristics, such as rural dirt roads, narrow paths, and low visibility, which effectively makes up for the shortcomings of urban public datasets in rural road modeling.

The experimental training parameter settings are shown in Table 1. The running environment is the Pytorch 1.7.0 framework based on Python 3.7, using four NVIDIA RTX 3090-24G GPUs, and the computing platform is CUDA 11.3. In order to speed up the training speed and improve the performance of the model, the model was encapsulated as Distributed Data Parallel (DDP) parallel training mode. In the experiment, Adam was used as the optimizer, the learning rate of the generator was set to 0.001, the learning rate of the discriminator was set to 0.0001, the batch size was set to 8, the coefficient λ of the penalty term was set to 10, and the total training iterations were 2000 kimg. The input and output dimensions of the image are 1024 × 1024.

### 3.2. Comparative Experiment Design

#### 3.2.1. Comparison of Different GAN Models

To verify the generation performance of the StyleGAN-ALL network, we compare it with DiffAugment-GAN [27] and CycleGAN, two representative networks for comparative experiments. The inception score (IS) and Fréchet inception distance (FID) of the generated images were calculated using the same training data and configuration. These two networks are chosen because DiffAugment-GAN maintains good image quality when the amount of data is limited by introducing a differentiable data augmentation module, which is suitable for the validation of small and medium-sized synthetic data generation tasks in this study. However, CycleGAN learns the mapping relationship between two different image domains to achieve style transfer, which is completely different from the mechanism of the StyleGAN series that uses added noise to generate images. The comparison of the two models helps to understand the impact of different generation mechanisms on the quality, diversity, and usability of generated images in downstream instance segmentation tasks and provides multiple perspectives for the study of image generation mechanisms.

#### 3.2.2. Comparative Experiments on Generated Images in Instance Segmentation Tasks

To further verify the usability of the synthesized images from the proposed StyleGAN-ALL generative model in downstream tasks, we design a comparative experiment of instance segmentation. A total of 100 manually labeled synthetic images are mixed with the real training dataset, and the performance is compared with the model trained only with real data. In the case of small data volume, we test whether the synthetic image can be effectively used by the existing instance segmentation model without negative impact on the model performance. This experiment not only focuses on accuracy improvement but also focuses on testing the compatibility and stability of generated images in actual tasks, which provides a basis for the practicability of generated data in perception tasks. In this study, four instance segmentation models, Mask R-CNN, SOLOv2, YOLOv8n, and OneFormer, are selected to train and evaluate the performance of the original dataset and the enhanced dataset, respectively, to verify the improvement effect of data enhancement methods on model performance.

The main reasons for choosing these four models are as follows: Mask R-CNN is a representative of the two-stage method. It is an instance segmentation framework based on the region proposal network (RPN) and fully convolutional networks (FCNs). High sensitivity to data quality (since its RPN relies on accurate proposal annotations). On the basis of Faster R-CNN [28], Mask R-CNN adds a branch network to predict the pixel-level semantic segmentation mask, which can achieve accurate segmentation of each object instance. SOLOv2 is an advanced paradigm for single-stage methods that specifically focuses on directly predicting a segmentation mask for each object instance, and the mask prediction mechanism responds strongly to the edge quality of the generated data. Building on the SOLO framework, SOLOv2 introduces two key improvements for mask detection and runtime efficiency: (1) mask learning, which enhances the ability to learn segmentation masks; and (2) maskNMS, which proposes matrix NMS to significantly reduce forward inference time and improve inference speed. YOLOv8n is a real-time model with lightweight architecture, inheriting the fine tradition of the YOLO series, and its parameter efficiency makes it easier to expose the impact of data noise. Moreover, the lightweight network architecture is adopted to achieve efficient real-time object detection on resource-limited devices such as mobile devices. The YOLOv8n framework is not limited to object detection but can also be extended to various vision tasks, including image classification and instance segmentation, and supports deployment on multiple hardware platforms. OneFormer is a representative model based on the transformer architecture, which is based on the multi-layer hierarchical vision transformer and has powerful context modeling capabilities. At present, SOTA performance is achieved in multiple image segmentation tasks. Using this model enables a more comprehensive assessment of the validity and compatibility of images generated by the improved StyleGAN2-ADA model compared to the CNN architecture of the previous three methods. Through these comparative experiments, the performance of different datasets across various models can be evaluated, thereby validating the improvement in data quality. Additionally, the contribution of data generated by the improved generative adversarial network to the model’s generalization ability can be assessed, providing robust data support for future research and practical applications.

### 3.3. Ablation Experiment Design

#### 3.3.1. DMN Ablation Experiment

In order to directly verify the effectiveness of the decoupling ability of the DMN module, four groups of control experiments are designed in this section, and StyleGAN2-ADA is used as the baseline to construct four different groups of FC combinations:

Model 1-1 is the original StyleGAN2-ADA.

Model 1-2 is to randomly group FC layers.

Model 1-3 is the design scheme of DMN by dividing the original eight-layer FC into four groups with two layers in each group.

Model 1-4 is the division of FC into two groups with four layers each, which is used to compare the influence of the number of groups on the coupling and generation effects.

#### 3.3.2. Verification Experiment of the Combined Effect of DMN and CCTB

In this ablation experiment, we aim to explore the influence of the decoupled mapping network and the CCTB module on the performance of the StyleGAN2-ADA network in generating rural road scene images. In this paper, StyleGAN2-ADA is used as the baseline model, and four variant models with different configurations are constructed:

Model 2-1 is the original StyleGAN2-ADA.

Model 2-2 (StyleGAN-4FC): added a decoupled mapping network based on Model 1.

Model 2-3 (StyleGAN-CCTB): adds the CCTB module based on Model 1.

Model 2-4 (StyleGAN-ALL): adds decoupling mapping network and CCTB modules on the basis of Model 1.

### 3.4. Data Evaluation Metrics

The data evaluation experiment is an image denoising algorithm experiment based on hybrid attention feature extraction and high-low frequency decomposition. The experimental dataset is divided into the original dataset and the synthetic dataset after data augmentation. The original dataset contains 1285 real images. The synthetic dataset is an experimental dataset composed of 1385 images after annotation and merging with real images. Through a series of experiments and training, it is verified that the images generated by the generative network have a significant role in improving the segmentation efficiency of the final instance segmentation model, which not only improves the segmentation accuracy of the model but also enhances its generalization ability.

#### 3.4.1. Data Augmentation Evaluation Metrics

In the current research, the quality assessment of generated images is mainly carried out from two aspects: qualitative assessment and quantitative assessment. Qualitative assessment relies on the subjective judgment of observers, giving scores through visual observation and similarity evaluation, and finally obtaining an overall index, but this way is highly subjective. In contrast, quantitative evaluation provides a more objective method, and commonly used quantitative evaluation methods include inception score (IS) [29] and Fréchet inception distance (FID) [30]. In this experiment, IS and FID are selected as the main evaluation metrics in order to objectively evaluate the quality of the generated images.

IS objectively evaluates the quality and diversity of image generation and uses the pre-trained Inception model to calculate the Kullback–Leibler [31] divergence between the marginal distribution and the conditional distribution. The calculation formula is shown in Formula (14).

(14) $$IS\left(G\right)=exp\left\{E_{x\sim p_{g}}D_{KL}\left[p\left(y|x\right)∥p\left(y\right)\right]\right\}$$

where $x∼P_{g}$ is the generated image, and $D_{KL}$ is the KL divergence. A higher IS score, that is, a higher KL value, indicates that the generated images are of higher quality and more diverse.

FID is to calculate the distance between the feature vector of the real image and the generated image. The smaller the FID value, the closer the generated image is to the real image, and the better the quality of the generated image. The basic idea of FID is to use the convolutional feature layer of the Inception network as a feature function φ, and use the feature function to model the true data distribution $P_{r}$ and the generated data distribution $P_{g}$ as two multivariate Gaussian random variables. In this way, the mean $\mu_{x}$, $\mu_{g}$ and the variance $\sum_{x}$, $\sum_{g}$ of the multivariate Gaussian distribution can be calculated. The calculation formula is given in Formula (15).

(15) $$FID\left(X,G\right)={∥\mu_{x}-\mu_{g}∥}_{2}^{2}+Tr\left(\sum_{x}+\sum_{g}-2{\left(\sum_{x}\sum_{g}\right)}^{\frac{1}{2}}\right)$$

where $\mu_{x}$ is the mean value of real image features; $\mu_{g}$ is the mean value of the generated image feature; $\sum_{x}$ is the variance of real image features; and $\sum_{g}$ is the variance of the generated image features.

#### 3.4.2. Model Performance Evaluation Metrics

In order to evaluate the specific impact of data augmentation on model performance, four models, Mask R-CNN, SOLOv2, YOLOv8n, and OneFormer, are trained experimentally using the original dataset and the StyleGAN-ALL enhanced dataset, respectively. At the same time, a comprehensive model performance evaluation system is constructed, and the key indicators adopted are shown in Table 2.

## 4. Results and Analysis

### 4.1. Data Augmentation Results Analysis

**Figure 7:**
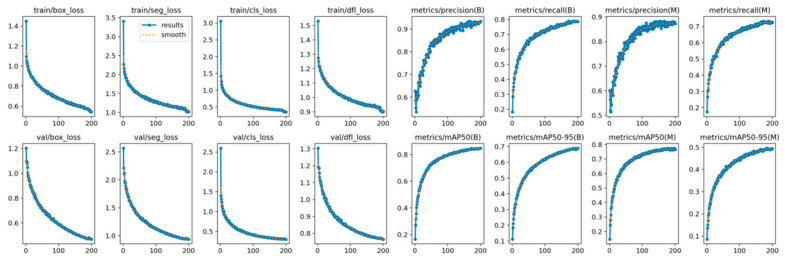
Loss and trend diagram of various metrics.

Figure 7 shows the trends of the loss functions and evaluation metrics during the model’s training process. As shown in the figure, during the initial training phase (the first 50 epochs), the loss functions (trainbox_loss and valbox_loss) decrease rapidly, while the evaluation metrics (metrics/precision(B), metrics/recall(B), and metrics/mAP50(B)) increase significantly. This indicates that the model can quickly learn features from the data and improve performance in the early stages. However, after 50 epochs, the changes in the loss functions and evaluation metrics gradually stabilize, entering a plateau. Finally, both the loss functions and evaluation metrics reach convergence, indicating that the model has learned a relatively stable solution on the training data.

**Figure 8:**
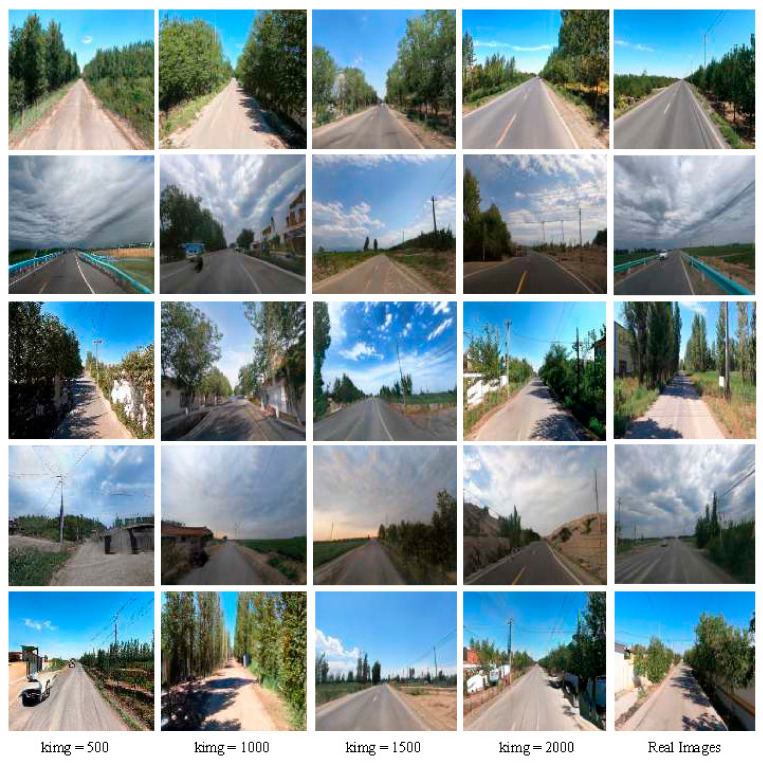
Iterative training effect diagram.

In order to comprehensively show the generation effect of the rural road scene in different iteration stages, Figure 8 shows where kimg represents the number of iteration cycles of model training. When kimg is 500, the generated images show obvious distortion problems: distorted road shapes, unclear boundaries between trees and sky, and blurred vehicle shapes, showing that the algorithm is still in the early stage of optimization. As training progresses to kimg 1000, significant improvements are observed: unnatural distortions in the road are greatly reduced, lane markings on the road surface become distinct, although vegetation details along the roadside remain slightly blurred, and the boundary between trees and the sky requires further refinement. This indicates that the generation quality is steadily improving. At kimg 1500, the realism of the images improves significantly: the boundary between the sky and trees becomes clearly distinguishable, details such as utility poles are finely rendered, and the diversity of environmental elements increases notably, reflecting the generative model’s enhanced ability to handle complex scenes. Finally, when iterating to kimg 2000, the generated rural road image is almost realistic; not only are the details of the road and the surrounding environment highly restored, but also the roadside buildings are constructed. This process not only shows the trajectory of the improved StyleGAN-ALL model gradually overcoming distortion and improving generation quality through iterative training but also highlights its excellent potential for image synthesis of complex scenes.

### 4.2. Comparative Experiment Results Analysis

#### 4.2.1. Comparison of Experimental Results with Different GAN Models

The comparative experimental results based on DiffAugment-GAN, CycleGAN, and StyleGAN-ALL models are shown in Table 3. The IS of DiffAugment-GAN and CycleGAN were 63.28 and 38.43, respectively, which were significantly lower than our proposed StyleGAN-ALL (IS = 77.31). And the FIDs were 17.54 and 34.76, respectively, which were also much higher than that of StyleGAN-ALL (FID = 12.42), indicating that there was a significant gap between the quality and diversity of images.

Experimental results show that StyleGAN-ALL is significantly better than other comparison methods in both indicators. CycleGAN has the lowest IS and FID, reflecting its poor adaptability in the generation task of this study. This may be because CycleGAN focuses more on style transfer in the image domain than on the generation of high-quality images, and its generated results are obviously deficient in structure and details. Although DiffAugment-GAN shows certain advantages in small sample scenarios, it is still slightly inferior in image quality evaluation indicators due to its relatively simple model architecture and lack of deeper feature modeling capabilities. In contrast, StyleGAN-ALL combines multiple improvement strategies, which not only improve the realism of the generated images (significant increase in IS value) but also achieve better performance in structural consistency (significant decrease in FID value). This fully demonstrates that the improved method proposed in this study has higher effectiveness and generalization ability in image generation tasks, which provides a solid foundation for the application of synthetic data in downstream vision tasks.

#### 4.2.2. Comparative Experimental Results of Generated Images in Instance Segmentation

In this study, Mask R-CNN, SOLOv2, YOLOv8-Segment, and OneFormer algorithms are used for comparison to evaluate the impact of data generated by adversarial generative networks on instance segmentation tasks. The study trained these four instance segmentation models using both the original dataset and the synthetic dataset and objectively compared their performance in instance segmentation tasks to explore the enhancement effect of the generated data on model performance. The comparison of the mean Average Precision (mAP) metrics for bounding box detection (B) and instance segmentation (M) for these four models on the original and synthetic datasets is shown in Table 4.

By comparing the performance of these two different data conditions, for the Mask R-CNN algorithm, the synthetic data improves the performance by 0.92 on mAP50(B) and 0.81 on mAP50(M) compared with the original data. Significant improvements are also observed in mAP50-95(B) and mAP50-95(M), two metrics that span multiple IoU thresholds. These improvements demonstrate that synthetic data not only enhances the detection performance of Mask R-CNN at higher confidence levels but also improves its stability across different IoU thresholds. For the SOLOv2 algorithm, although the performance improvement from synthetic data is relatively small, increases are observed in mAP50(B), mAP50(M), mAP50-95(B), and mAP50-95(M). This indicates that even for the already high-performing SOLOv2 algorithm, synthetic data can still provide a positive impact to some extent. For the YOLOv8n algorithm, the synthetic data bring a 0.71 improvement on mAP50(B), and there is also a certain increase on Map50-95(B). Although the improvement on mAP50(M) and Map50-95(M) is small, it still proves the positive impact of synthetic data on the performance of the YOLOv8n algorithm as a whole. For the OneFormer algorithm, the highest mAP50(B) and mAP50(M) are achieved after using the synthetic dataset, which are 0.74 and 0.76 higher than those of the original dataset, respectively, indicating that the generated images are also compatible with the new architecture model. Based on the analysis of the experimental data, it is observed that the synthetic data has a positive impact on the performance indicators of different algorithms.

In addition, this study applies the three aforementioned instance segmentation algorithms to both the original and synthetic datasets, comparing their precision and recall in the instance segmentation task. The results are presented in Table 5.

For the Mask R-CNN algorithm, the precision(B) of using synthetic data is slightly improved compared with the original data, increasing from 69.15 to 69.25. For precision(M), synthetic data provide a more significant improvement, increasing from 65.04 to 67.28. Additionally, synthetic data improves recall(B) and recall(M) by 1.15 and 1.93, respectively. These results demonstrate that synthetic data enhances both precision and recall for the Mask R-CNN algorithm, with particularly notable improvements in object segmentation. For the SOLOv2 algorithm, the use of synthetic data brings a significant improvement in precision(B), from 88.96 to 91.44, an increase of 2.48 percentage points. For precision(M), although the improvement is smaller, it increases from 84.42 to 85.56. In terms of recall, synthetic data also has a positive impact, improving recall(B) and recall(M) by 1.02 and 1.21, respectively. These results demonstrate that training the SOLOv2 algorithm with synthetic data can also lead to significant performance enhancements. For the YOLOv8n algorithm, although the improvements in precision(B) and precision(M) from synthetic data are relatively small, they increase from 92.86 to 93.44 and from 87.76 to 88.16, respectively. However, in terms of recall, synthetic data provides more significant improvements, with recall(B) and recall(M) increasing by 0.96 and 1.09, respectively. This demonstrates that synthetic data has a particularly notable impact on improving recall for the YOLOv8n algorithm.

For the OneFormer algorithm, the introduction of synthetic data brings a positive improvement in all indicators. In terms of precision(B), it increased from 93.52 of the original data to 94.65, an increase of 1.13 percentage points. The precision(M) is also improved from 88.75 to 89.64, with an increase of 0.89. At the same time, the synthetic data brings 1.02 and 1.23 improvements in recall(B) and recall(M), respectively. Recall(B) increases from 78.86 to 79.88, and recall(M) increases from 73.19 to 74.42. These results show that the synthetic image not only further enhances the segmentation performance of OneFormer in terms of accuracy but also improves its ability to identify and cover the object, especially in the segmentation of complex or detailed regions, which shows stronger stability and robustness.

Combined with the experimental results, the proposed StyleGAN-ALL generates images with good generalization ability and can provide valuable supplementary data for the current mainstream advanced instance segmentation algorithms. In several mainstream instance segmentation models, the introduction of annotated generated images does not cause performance degradation and even achieves a small performance improvement on many models. This shows that the images generated by StyleGAN-ALL have good realism in feature distribution and semantic structure and can be used as high-quality supplementary data to participate in model training. Although the improvement is limited, the performance is stable under the condition of a small amount of data, which proves the effectiveness and security of the synthesized image. This result lays the foundation for subsequent large-scale generated data applications and also validates the potential of generative models to assist sensing tasks.

**Figure 9:**
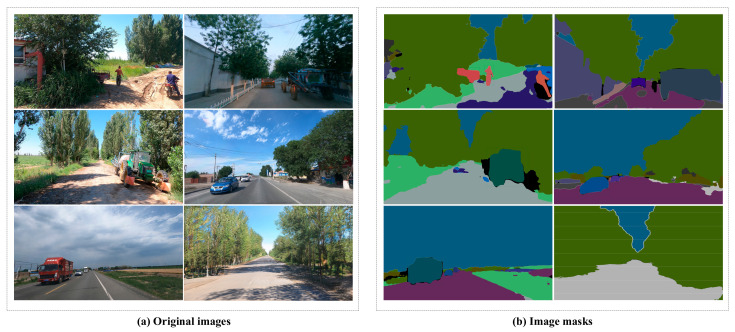
Visualization of OneFormer model.

To show the effect of the data augmentation technique more intuitively, the OneFormer model with the highest training accuracy is used to perform inference on the synthetic dataset, as shown in Figure 9. Figure 9a is the original image in the dataset, and Figure 9b is the object detection box and instance segmentation result corresponding to the original image on the left. The object detection box is green, and the individual instances are distinguished by different colors. Figure 9 shows that the model can segment the instances in the scene well, and there are few false detections and missed detections, which further verifies the effectiveness of synthetic data in improving the segmentation performance of the model.

### 4.3. Ablation Experiment Results Analysis

In order to verify the improvement of the network performance by the two improvements in this paper, two sets of ablation experiments are designed to evaluate the impact of these improvements on the model.

#### 4.3.1. Results of the DMN Ablation Experiment

In this experiment, four different FC grouping methods were designed: a group of eight-layer FCs, a random stratified FC, a four-group FC, and a two-group FC, respectively. The effectiveness of the DMN design was verified by IS, FID, and mutual information (MI) indicators, and the experimental results are shown in Table 6.

From the above table results, it can be seen that the grouping strategy has a significant impact on the model performance and decoupling. The baseline model (Model 1-1) achieves an MI of 0.38, which reflects the inherent coupling of the latent code, while the MI of the random grouping (Model 1-2) further rises to 0.47, indicating that disordered grouping will exacerbate feature entanglement. In contrast, the four-group design (Model 1-3) shows a great advantage, and its MI is 0.12, which is 0.26 lower than the original model. At the same time, the generation quality of the proposed model IS optimal, and the IS and FID are 48.27 and 23.78, respectively, which proves that the feature representation is effectively separated by structured grouping. While grouping two (Model 1-4) shows a certain decoupling effect, with an MI of 0.28, which is 0.10 lower than that of the original model, the performance improvement is limited, especially since the FID value has little difference, which verifies the rationality of our DMN design grouping four, which significantly reduces coupling while maintaining generation ability.

#### 4.3.2. Experimental Results of the Combined Effect of DMN and CCTB

Firstly, StyleGAN2-ADA is selected as the baseline model. On this basis, two kinds of improvements are implemented, respectively: one is to add DMN, and the other is to introduce the CCTB module. Finally, the StyleGAN-ALL model is created by combining these two improvements. Through qualitative and quantitative evaluation and comparison, the effectiveness of the proposed improvement measures is verified.

**Figure 10:**
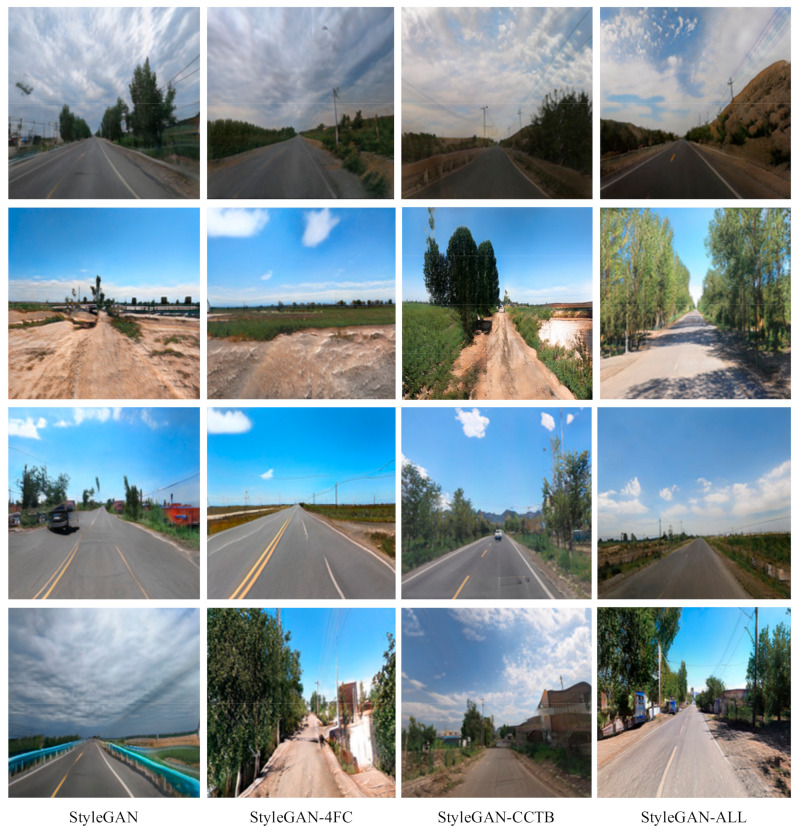
Visualization of ablation experiment results.

The qualitative evaluation results are shown in Figure 10. Through qualitative and quantitative evaluation of the four models, the following results were observed: the original Model 2-1 generates images with many redundant objects randomly scattered in the sky, highly curved lane lines, and a limited variety of generated objects. Model 2-2 performs better in reducing image distortions, but the scene composition is relatively simple, the generated road texture appears unreal, and buildings such as houses are severely deformed. Model 2-3 demonstrates improved category diversity, capable of simulating objects such as vehicles and clouds, but it still struggles with complex features, and buildings such as houses exhibit some deformation. Model 2-4 shows significant improvements in image quality, edge clarity, and scene complexity. The generated lane lines are straight, the sky contains no excess vegetation, and the outlines of houses are clear and free from distortion. This demonstrates the synergistic effect of combining the two modules. Ultimately, the experimental results reveal the specific contributions of each module to model performance, providing guidance for further optimization.

To more objectively evaluate the image generation performance of each network model, this study compared the inception score (IS) and Fréchet inception distance (FID) of the four models, with the results shown in Table 7. By comparing these metrics, the performance of the models in terms of image generation quality can be comprehensively assessed, thereby validating the effectiveness of the proposed improvements.

According to the data in Table 7, it can be observed that Model 2-4 (StyleGAN-ALL) exhibits significant advantages in two key evaluation metrics: IS and FID. Specifically, Model 2-4 achieves an IS value of 77.31, significantly higher than Model 2-1 (42.38) and Model 2-2 (48.27), indicating a substantial improvement in the quality of generated images. In addition, the FID value of Model 2-4 is reduced to 12.42, which is also significantly lower than that of Model 2-3 (16.00) and Model 2-1 (25.09), which further confirms the superior performance of StyleGAN-ALL in terms of the realism and diversity of generated images. These results demonstrate that by integrating the DMN and the CCTB module, StyleGAN-ALL achieves significant performance improvements in image generation tasks, highlighting the effectiveness of these enhancements and their positive impact on model performance.

## 5. Conclusions

Currently, autonomous navigation technology for agricultural machinery in rural road environments is becoming a hot research topic. Constructing a rural road image dataset provides essential data support for developing and enhancing the safety of intelligent agricultural machinery navigation systems. To expand the dataset and improve the diversity of sample images, this study proposes an improved StyleGAN-ALL network model based on StyleGAN2-ADA. This model reduces the coupling of latent space encodings by introducing a decoupled mapping network and enhances the model’s ability to capture complex contextual information and spatial layouts through the convolutional coupling transfer block. This approach not only addresses the gap in data generation for rural road scenarios but also significantly improves the effectiveness of data augmentation by proposing and combining the DMN and CCTB modules. It provides robust technical support and data-driven foundations for training instance segmentation models in autonomous agricultural machinery systems operating in rural road environments.

Through ablation experiments, the IS and FID metrics of the model before and after improvements were compared. The experimental results show that the improved network achieved an 82% increase in IS and a 50% reduction in FID, indicating a significant enhancement in the quality and realism of the generated images. Additionally, by comparing the performance of Mask R-CNN, SOLOv2, YOLOv8n, and OneFormer on the original dataset and the synthetic dataset augmented by StyleGAN-ALL, the significant role of the generative network in improving the segmentation efficiency of instance segmentation models was further validated. This not only enhances the segmentation accuracy of the models but also improves their generalization capabilities.

The synthetic data generated in this study show good transfer ability and generalization in the instance segmentation task, which can have the potential to reduce the cost of real data labeling to a certain extent, and provide cost-effective training resources for agricultural autonomous driving systems. In the future, this research will further optimize the generative model, particularly focusing on improving the clarity and detail representation of small objects in images. This will further enhance the performance and reliability of agricultural machinery navigation systems in rural road environments, thereby providing stronger data support and technical guarantees for the development of intelligent agricultural machinery technology.

## Figures and Tables

**Table 1:** Model training parameter settings.

Parameter Name	Description	Value
batch size	Size of a single batch	8
learning rate-generator	Learning rate for the generator	0.001
learning rate-discriminator	Learning rate for the discriminator	0.0001
kimg	Number of iterations	2000
beta	Exponential decay rate for the optimizer	0.5

**Table 2:** Model performance evaluation metrics.

Metric Name	Description
mIoU (Intersection Over Union)	Measure the average of the degree of overlap between the predicted boundary and the true boundary.
Precision	The proportion of correctly predicted positive samples among all predicted positives.
Recall	The proportion of correctly predicted positive samples among all actual positives.
AP (Average Precision)	The area under the precision–recall curve at different thresholds.
mAP (Mean Average Precision)	The mean of average precision (AP) across all categories is used to evaluate the model’s overall recognition performance.
Boundary Displacement Error (BDE)	The average distance between predicted boundaries and ground truth boundaries.

**Table 3:** Comparison of experimental results with different GAN models.

Model	IS	FID
DiffAugment-GAN	63.28	17.54
CycleGAN	38.43	34.76
**StyleGAN-ALL**	**77.31**	**12.42**

**Table 4:** Comparison of mAP metrics for bounding box detection (B) and instance segmentation (M) on original and synthetic datasets.

Data	Algorithm	mAP50 (B)	mAP50 (M)	mAP50-95 (B)	mAP50-95 (M)
Original	Mask R-CNN	54.92	49.26	38.79	28.60
**Synthetic**	**Mask R-CNN**	**55.84**	**50.07**	**39.23**	**29.08**
Original	SOLOv2	78.56	71.53	60.08	43.59
**Synthetic**	**SOLOv2**	**78.76**	**71.93**	**60.15**	**43.79**
Original	YOLOv8n	84.13	77.12	68.64	49.40
**Synthetic**	**YOLOv8n**	**84.84**	**77.24**	**68.96**	**49.52**
Original	OneFormer	85.79	77.89	70.08	50.64
**Synthetic**	**OneFormer**	**86.53**	**78.65**	**71.14**	**51.39**

**Table 5:** Comparison of precision and recall metrics for instance segmentation models.

Data	Algorithm	Precision (B)	Precision (M)	Recall (B)	Recall (M)
Original	Mask R-CNN	69.15	65.04	49.81	44.89
**Synthetic**	**Mask R-CNN**	**69.25**	**67.28**	**50.96**	**46.82**
Original	SOLOv2	88.96	84.42	70.63	65.78
**Synthetic**	**SOLOv2**	**91.44**	**85.56**	**71.65**	**66.99**
Original	YOLOv8n	92.86	87.76	77.81	71.61
**Synthetic**	**YOLOv8n**	**93.44**	**88.16**	**78.77**	**72.70**
Original	OneFormer	93.52	88.75	78.86	73.19
**Synthetic**	**OneFormer**	**94.65**	**89.64**	**79.88**	**74.42**

**Table 6:** DMN ablation experiments in different groups.

Model	IS	FID	MI
Model 1-1	42.38	25.09	0.38
Model 1-2	42.59	25.53	0.47
**Model 1-3**	**48.27**	**23.78**	**0.12**
Model 1-4	44.35	24.94	0.28

**Table 7:** Ablation experiments with combined effects of DMN and CCTB module.

Model	IS	FID
Model 2-1	42.38	25.09
Model 2-2	48.27	23.78
Model 2-3	74.89	16.00
**Model 2-4**	**77.31**	**12.42**

## Data Availability

Data are available upon request.
